# History of Medicine

**Published:** 1911-09

**Authors:** 


### Reviews of Books.

**History of Medicine.** By Dr. MAX NEUBURGER. Translated by ERNEST PLAYFAIR, M.B., M.R.C.P. Vol. I. Pp. x, 404. London: Oxford Medical Publications. 1910.

Of the many valuable works which the directors of the Oxford Medical Publications have brought out, Neuburger's *History of Medicine* stands in the first rank. Books such as this have only too often remained untranslated, and therefore inaccessible to those who do not combine linguistic ability with historical cravings. There is an encyclopædic wealth of information in this first volume, which deals with medicine from prehistoric times to the Renaissance. The scanty vestiges of prehistoric medicine are elucidated and amplified by many illustrations drawn from the habits and customs of contemporary savage man, who in all save chronology is truly "prehistoric."

Thence the author passes in critical survey through the medicine of the East, including the healing art of Mesopotamia, Ancient Egypt, Persia, the Old Testament, India, China and Japan. Next Classic Antiquity comes under review, rich in names of physician-philosophers and philosophic schools, which presently give place to the barren centuries of the decline of Rome. Finally the volume ends with the romance of medicine in the Middle Ages, when philosophy, and with it medicine, wandered in the wilderness like some nomadic Arab tribe. Driven from Byzantium and Alexandria to Syria, thence with Nestorianism from Edessa to Persia and back with the conquering hosts of Islam to the Mediterranean shores, through North Africa to Spain, to Sicily and to Italy, the seeds of our modern philosophy and science were sown broadcast, some to wither on stony ground, some in the lapse of centuries to bear the harvest which our own age has scarcely yet discerned. These were the centuries when medicine itself made little progress until the spirit of science should have cleared the way of progress. Though in Neuburger's words, "Just as occidental Moslem philosophic thought was already imbued with the first breath of that free spirit which was later to sweep over the whole of Western Europe, so did Hispano-Arabic medicine—at least as it took shape in the hands of its worthiest representatives—manifest a certain leaning towards scientific research, a notable tendency in favour of open-minded clinical observation, hand in hand with sceptical rejection of unverified tradition."

So the volume ends, with the beginning of Arabic influence upon occidental medicine and occidental thought, leaving for the second volume the story of the Renaissance and the commencement of modern times. We cannot criticise the book, we can only revel and admire. Sometimes we miss the comparative touch, we want to be reminded of how the world revolved while medicine was in the making. Has medicine progressed with social evolution ? Did it rise and fall with monarchies and republics ? How fared it amid the clash of arms or babbling voices of law-makers ? Or did it and does it still stand aloof, looking on the struggles of the human race for existence with scientific detachment, self-centred and untouched ?

These are no shortcomings in Professor Neuburger's splendid history. He reminds us of our heritage from the ages, until, with Emerson, we feel that " man is explicable by nothing less than all his history," and we want to claim to be freemen of the whole estate. Neuburger writes, indeed, of medicine, but he cannot fetter the thoughts of his readers, and thus, perhaps, his science becomes an art.

